# Reimbursement Design and Procedure‐Level Gross Profit of Robot‐Assisted Gastrectomy in a Publicly Financed Health System: Evidence From Japan

**DOI:** 10.1002/hpm.70127

**Published:** 2026-08-17

**Authors:** Yoshimasa Akashi, Koichi Ogawa, Yohei Owada, Tsuyoshi Enomoto, Kinji Furuya, Kazuhiro Takahashi, Osamu Shimomura, Yoshihiro Miyazaki, Tatsuya Oda

**Affiliations:** ^1^ Department of Gastrointestinal and Hepato‐Biliary‐Pancreatic Surgery Faculty of Medicine University of Tsukuba Tsukuba Ibaraki Japan

**Keywords:** gastric cancer surgery, health economics, hospital profitability, Japanese national health insurance, reimbursement policy, robotic surgery

## Abstract

Publicly financed healthcare systems must balance technological innovation with fiscal constraints. Reimbursement mechanisms influence institutional decisions regarding the adoption of high‐cost surgical technologies. This study evaluated whether current reimbursement designs adequately reflect procedure‐level gross surgical profit of robot‐assisted gastrectomy within the Japanese National Health Insurance system. A retrospective cost analysis was conducted for curative gastric cancer surgery performed at a tertiary institution in 2024. Gross surgical profit was defined as total reimbursement minus procedure‐related costs, including consumables, robot maintenance, and labour costs. Postoperative complication‐related costs and total episode‐of‐care costs were not incorporated into the analysis. Fifty‐two gastrectomies were analysed. Open and laparoscopic distal gastrectomy generated positive gross profits, whereas robotic distal gastrectomy resulted in a negative gross margin (−¥87,075 per case). Although robotic total gastrectomy showed a positive gross profit in absolute terms, it remained substantially less profitable than open total gastrectomy. Increased procedure‐related costs associated with robotic surgery were only partially offset by higher reimbursement. Sensitivity analyses indicated that gross profit was more responsive to changes in surgical fees and material costs than to maintenance or labour costs. These findings suggest that current reimbursement increments may not fully reflect procedure‐related costs associated with robotic gastrectomy and highlight the importance of procedure‐specific reimbursement evaluation when introducing high‐cost surgical technologies into publicly financed healthcare systems.

## Highlights

- Robotic distal gastrectomy shows negative gross profits.
- Gross profit patterns differ substantially across surgical approaches.
- Current reimbursement increments only partially offset robotic costs.
- Procedure‐specific reimbursement evaluation is important for high‐cost surgical technologies.

## 1 Introduction

Publicly financed healthcare systems increasingly face the challenge of balancing technological innovation with financial constraints. Robot‐assisted surgery has rapidly expanded worldwide because of its potential to improve surgical precision, enhance visualisation, and reduce surgical invasiveness [1, 2, 3]. Robotic systems provide high‐definition three‐dimensional vision, improved ergonomics, and articulated instruments that allow surgeons to perform complex procedures in confined anatomical spaces with greater dexterity [4, 5]. These technological advantages have contributed to the widespread adoption of robotic surgery across multiple surgical specialties and are often associated with improved perioperative outcomes, such as reduced complications and faster recovery [3, 6, 7]. However, the rapid diffusion of robotic surgery has also raised concerns regarding its economic implications for healthcare systems and hospital finances. Previous studies have emphasised that while robotic surgery may improve clinical outcomes, the high acquisition costs of robotic platforms, ongoing maintenance contracts, and the use of dedicated disposable instruments substantially increase procedural costs and may place financial pressure on healthcare providers [3, 4, 5, 8].

Economic evaluations of robotic surgery have produced heterogeneous findings depending on the procedure type and analytical perspective. Numerous studies have demonstrated that robotic surgery is associated with significantly higher intraoperative costs than conventional laparoscopic or open surgery, due to equipment acquisition, maintenance, and consumable instrument expenses [8, 9, 10]. Simultaneously, robotic surgery may reduce downstream healthcare costs through shorter hospital stays, lower complication rates, and decreased use of intensive care resources. For example, studies evaluating robotic cystectomy and other complex procedures have suggested that improved perioperative outcomes may partially offset higher operative costs when broader health outcomes, such as quality‐adjusted life years (QALYs), are considered [3, 6, 7]. In addition, hospital surgical volume has been shown to influence cost efficiency because higher procedure volumes distribute fixed capital costs across a larger number of cases [3, 7].

Despite the growing body of economic evaluations, several methodological limitations remain. First, many studies have examined robotic surgery from the perspective of patients or healthcare payers, whereas the financial consequences for hospitals—including capital investment, depreciation of robotic systems, and service contracts—have been less frequently evaluated [4]. Second, many economic analyses rely primarily on short‐term outcomes, such as perioperative complications and length of hospital stay, while long‐term outcomes, including oncologic survival, long‐term quality of life, and extended healthcare utilization, remain insufficiently assessed [11, 12, 13]. Third, in several procedures, particularly colorectal surgery, robotic approaches have not consistently demonstrated clear oncologic or clinical superiority over conventional laparoscopic techniques, despite substantially higher procedural costs, raising ongoing concerns regarding value for money [12, 14].

The financial implications of robotic surgery also vary substantially across healthcare systems, depending on reimbursement structures and insurance design. In the United States, the diffusion of robotic surgery has been driven partly by hospital competition and marketing incentives, contributing to rapid adoption but also raising concerns regarding escalating system‐wide healthcare expenditures [15, 16]. In contrast, in countries where robotic procedures are not reimbursed by national health insurance, such as South Korea, patients frequently bear the full cost of robotic surgery, resulting in substantial out‐of‐pocket expenditures and potential inequities in access to advanced surgical technologies [12, 13, 14]. European publicly funded healthcare systems often apply explicit cost effectiveness thresholds when evaluating new technologies, and robotic procedures may face restrictions when the incremental cost effectiveness ratios exceed acceptable willingness‐to‐pay levels [6, 10].

Japan provides a distinctive context for evaluating the reimbursement structure and procedure‐related financial implications of robotic surgery. Since 2018, multiple robotic procedures have been gradually incorporated into the national health insurance system, leading to a rapid expansion in the use of robotic technology [2, 17]. However, for many procedures, the reimbursement level for robotic surgery remains similar to that for conventional laparoscopic surgery because clear clinical superiority has not been consistently demonstrated [4]. Gastric cancer surgery is one of the few procedures in Japan for which additional reimbursement for robotic surgery has been approved based on prospective clinical evidence demonstrating improved perioperative outcomes compared to laparoscopic surgery [18, 19]. Consequently, robotic gastrectomy receives a higher procedure fee than laparoscopic gastrectomy under the current national fee schedule. However, it remains unclear whether this reimbursement increment adequately reflects the increased procedure‐related costs associated with robotic gastric cancer surgery.

Therefore, this study aimed to evaluate the gross profit of curative gastric cancer surgery at a single institution and compare the financial outcomes of robotic, laparoscopic, and open surgical approaches. By examining procedure‐specific revenues and costs, this study explored how current reimbursement structures relate to procedure‐level gross surgical profit in robotic gastrectomy within a publicly financed healthcare system.

## 2 Methods

This study adopted a hospital‐provider perspective and evaluated intraoperative gross surgical profit over a one‐year period. This perspective focuses on procedure‐level institutional financial outcomes rather than societal cost effectiveness.

### 2.1 Data Collection

This study examined the annual gross surgical profit associated with gastric cancer surgery performed at Tsukuba University Hospital between January and December 2024. Because complete institutional records of non‐reimbursed material use were not systematically available for all cases, material costs were estimated using representative procedural material‐utilization patterns.

### 2.2 Surgical Procedure

At our institution, robot‐assisted surgery was generally the preferred minimally invasive approach for curative gastric cancer surgery during the study period. However, laparoscopic distal gastrectomy (DG) was performed because of the limited availability of robotic operating slots. A robotic approach was generally selected for total gastrectomy (TG) and proximal gastrectomy (PG). Open surgery was preferentially performed in cases of type 4 gastric cancer, large primary tumours (> 8 cm), remnant gastric cancer, or gastric cancer with suspected invasion of adjacent organs.

Representative reconstruction patterns were used for material‐cost estimation. For DG, Roux‐en‐Y reconstruction was selected because it was the most commonly performed reconstruction method across surgical approaches at our institution during the study period (Supporting Information S1: Table S1). For TG, costs were also calculated based on Roux‐en‐Y reconstruction.

### 2.3 Calculation of Material Costs

Material costs were estimated using representative procedural material‐utilization patterns for each surgical approach. Representative material sets were defined by the operating surgeons based on routine clinical practice at our institution because complete case‐level records of non‐reimbursed consumable use were not systematically available in the institutional database. Material‐cost estimation focused primarily on high‐cost consumables, typically costing 1000 JPY or more per item, including robotic instruments, stapling devices, barbed sutures, and disposable robotic accessories. Conventional low‐cost sutures were excluded because their relative contribution to total procedural costs was considered minimal.

Material costs were calculated based on institutional purchase prices provided by the procurement department. For reusable robotic instruments, the cost per use was estimated by dividing the purchase price by the manufacturer‐specified maximum number of uses. All reimbursement values and institutional cost estimates were based on the 2024 Japanese reimbursement schedule and institutional procurement prices and are expressed in nominal 2024 Japanese yen (JPY).

#### 2.3.1 Gross Surgical Profit

Gross surgical profit was calculated using the following formula:

Grosssurgicalprofit=(surgicalprocedurefee)+(reimbursablematerialcosts)−(materialcosts)−(robotmaintenancecosts)−(labourcosts).

Robot‐related institutional costs included annual depreciation of the robotic systems and maintenance‐contract expenses. Annual depreciation was calculated based on the institutional accounting schedule for two da Vinci Xi systems (Intuitive Surgical Inc.) installed at our hospital. For the primary analysis, total annual robot‐related costs were equally allocated across all robotic procedures performed during the study period (346 robotic procedures in 2024) to estimate a per‐case maintenance cost. Although operative‐time‐weighted allocation represents another possible accounting approach, accurate implementation would require operative‐time data for all robotic procedures across multiple specialties. Therefore, equal per‐case allocation was adopted as a pragmatic and transparent institutional accounting framework for the present analysis. Additional sensitivity analyses examining variations in robot maintenance costs were performed to evaluate the robustness of the findings under different accounting assumptions.

Labour costs were calculated based on the hospital's salary regulations and the estimated operation time. For each procedure, the labour cost was derived by multiplying the procedure‐specific operative duration by the standardized hourly wages for surgeons and nurses, assuming the participation of three surgeons and two nurses per operation.

### 2.4 Comparative Framework for Profitability Analysis

For the DG, profitability was compared across open, laparoscopic, and robotic approaches. For the TG, comparisons were limited to open and robotic approaches because no laparoscopic TG was performed during the study period. PG was excluded from comparative profitability analyses because no open or laparoscopic PG cases were available, precluding a meaningful comparison across surgical approaches.

### 2.5 Sensitivity Analysis

To examine the robustness of gross profit estimates, sensitivity analyses were performed for scenarios in which each revenue item (surgical fees and reimbursable material costs) and cost item (material costs, robot maintenance costs, and labour costs) associated with robot‐assisted DG and TG increased or decreased by 20%.

### 2.6 Ethics Statement

Approved by the Institutional Review Board of the University of Tsukuba Hospital (Approval No. R08‐013, May 26, 2026). The requirement for informed consent was waived because of the retrospective nature of the study. Information regarding the study was disclosed using an institutional opt‐out procedure.

## 3 Results

### 3.1 Case Distribution and Gross Profit of Gastric Cancer Surgery

During the study period, curative‐intent gastrectomy was performed in 56 patients, including 36 distal gastrectomy (DG), 16 total gastrectomy (TG), and four proximal gastrectomy (PG) cases. Of these, 52 DG and TG cases were included in the comparative profitability analysis. PG cases were excluded because no open or laparoscopic PG procedures were performed during the study period, precluding meaningful comparison across surgical approaches.

Of these procedures, 24 (46.2%) were performed using a robot‐assisted approach (18 DG and 6 TG), and 12 (23.1%) were performed laparoscopically (all DG) (Table 1). The remaining patients underwent open surgery. The distribution of surgical approaches differed by procedure type. Among the DG cases, robotic surgery was the most frequently performed approach, accounting for half of all DG procedures, whereas laparoscopic DG accounted for approximately one‐third of the cases. In contrast, TG was predominantly performed using the open approach, with robotic surgery used in a smaller proportion of patients.

**TABLE 1:** Clinical outcomes of all gastrectomy cases during the study period.

	Distal gastrectomy (DG)			Total gastrectomy (TG)			Proximal gastrectomy (PG)		
	Open	Lap	Robot	Open	Lap	Robot	Open	Lap	Robot
Number of cases	6	12	18	10	0	6	0	0	4
Operation time	315.5 ± 106.3	323.3 ± 99.7	407.3 ± 101.5	303.3 ± 91.1	N.A.	482.3 ± 84.2	N.A.	N.A.	433.0 ± 78.6
Blood loss	278.5 ± 152.6	42.8 ± 73.7	44.7 ± 41.9	676.0 ± 871.6	N.A.	92.7 ± 69.3	N.A.	N.A.	52.0 ± 73.9
Complication (≧ CD grade 3)	1 (16.7%)	2 (16.7%)	0	1 (10%)	N.A.	0	N.A.	N.A.	0
Postoperative hospital stay (day)	9.3 ± 1.8	12.7 ± 8.6	9.9 ± 2.2	12.2 ± 4.3	N.A.	8.5 ± 1.4	N.A.	N.A.	14.3 ± 6.3

Abbreviations: CD, Clavien‐Dindo classification; Lap, laparoscopic; N.A., not applicable.

Operative characteristics varied across surgical approaches. Robotic procedures were generally associated with longer operative times than open and laparoscopic procedures. However, the estimated blood loss was substantially lower in minimally invasive approaches than in open surgery. The incidence of major postoperative complications (Clavien–Dindo grade ≥ 3) remained relatively low across all approaches during the study period. These clinical outcomes are presented to provide contextual information regarding procedural characteristics across surgical approaches and were not incorporated into the gross surgical profit calculation.

The gross profit per case for each procedure and surgical approach is summarised in Table 2. Material costs for each procedure were calculated based on procedure‐specific material utilization derived from representative cases (Supporting Information S1: Table S2). Open and laparoscopic DG generated positive gross profit per case, whereas robot‐assisted DG resulted in a negative gross profit of ¥87,075 per case. TG generated positive gross profits with both open and robot‐assisted approaches; however, the gross profit associated with robot‐assisted TG was approximately one‐third of that observed with open TG.

**TABLE 2:** Gross profit and cost components per case according to surgical approach.

Surgical approach	Total reimbursement	Material cost	Maintenance cost	Labour cost	Total cost	Gross profit
(A) Distal gastrectomy (DG)						
Open	688,700	252,634	—	51,642	304,276	384,424
Laparoscopic	796,200	386,417	—	57,380	443,797	352,403
Robotic	890,900	594,433	311,243	72,299	977,975	−87,075
(B) Total gastrectomy (TG)						
Open	883,400	329,372	—	64,266	393,638	489,762
Robotic	1,228,500	664,125	311,243	87,218	1,062,586	165,914

*Note:* All values are expressed in 2024 Japanese yen (JPY).

Differences in gross profit across surgical approaches were primarily driven by variations in material and robot‐related maintenance costs. In DG, robotic surgery required substantially higher material expenditures because of the use of dedicated robotic instruments and additional consumables. When robot maintenance costs were allocated on a per‐case basis, the total procedural cost exceeded the reimbursed surgical fee, resulting in a negative gross profit for robotic DG.

In contrast, although robotic TG was also associated with higher material and maintenance costs than open TG, the higher reimbursement for this procedure partially offset the increased costs. Consequently, robotic TG remained profitable at the institutional level, albeit with a substantially smaller gross margin than open surgery.

### 3.2 Simulated Annual Gross Profit Under Different Surgical Approach Distributions

A simulation analysis was conducted to estimate the annual gross profit under different hypothetical distributions of surgical approaches, assuming a constant annual surgical volume.

As shown in Figure 1, the estimated annual gross profit was ¥21.7 million in a hypothetical scenario in which all cases were performed using the open or laparoscopic approach. Under the current distribution of surgical approaches, the estimated annual gross profit was ¥10.9 million. In contrast, a hypothetical scenario in which all cases were performed using a robot‐assisted approach resulted in no net positive gross profit.

**FIGURE 1:**
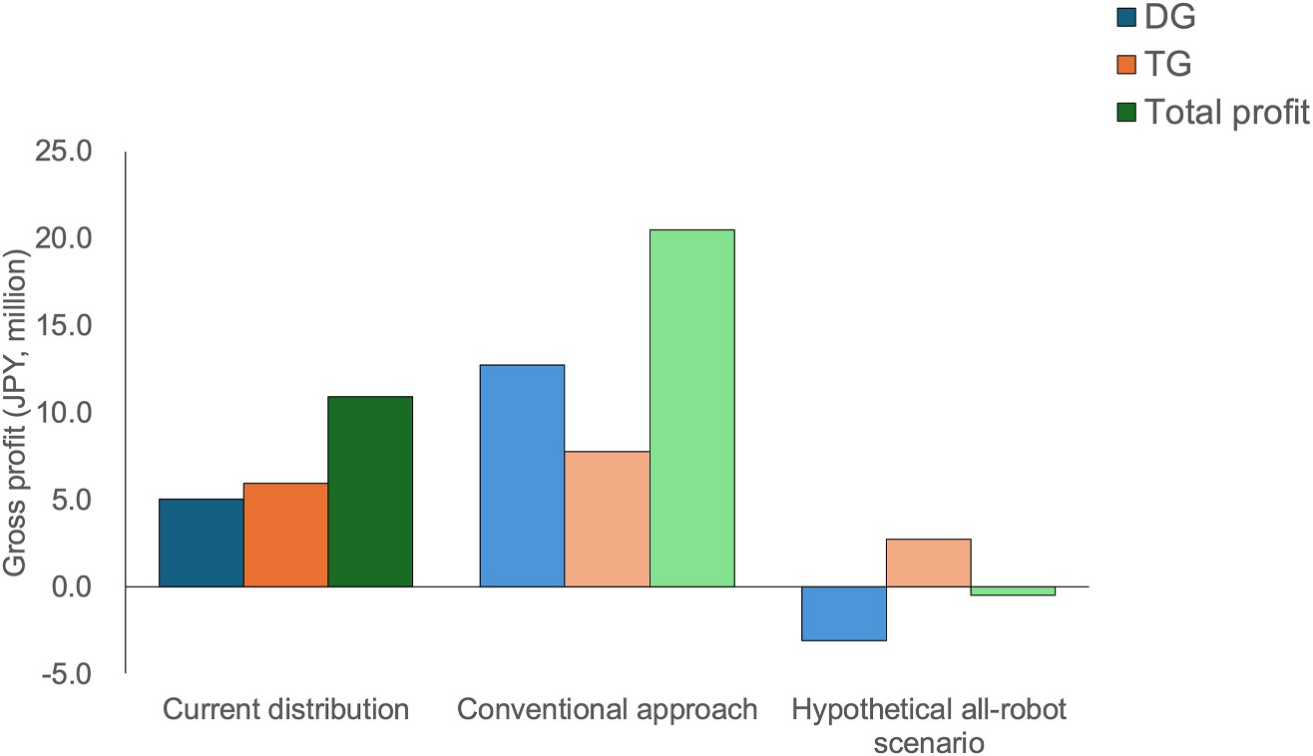
Simulated annual gross profit under different surgical approach distributions. Simulation of annual gross profit from gastric cancer surgery under different hypothetical distributions of surgical approaches, assuming a constant annual case volume. The ‘current distribution’ reflects the actual proportion of open, laparoscopic, and robotic procedures performed during the study period. The conventional approach scenario assumed laparoscopic distal gastrectomy and open total gastrectomy, whereas the all‐robot scenario assumed that all cases were performed robotically. All values are expressed in 2024 JPY.

A supplementary scenario analysis assuming a higher annual robotic case volume of 400 procedures was conducted to explore the potential impact of improved system utilization (Supporting Information S1: Figure S1). In many institutions, an annual volume of approximately 200 robotic procedures per system is commonly cited as a pragmatic benchmark for efficient utilization. Because two robotic systems were installed at our institution, a scenario of 400 procedures was used as a hypothetical upper‐bound estimate of achievable utilization.

Under this assumption, the maintenance cost per case decreased; however, the overall profitability changed only modestly. This finding suggests that even if robotic system utilization increases, volume expansion alone may be insufficient to resolve the structural imbalance between reimbursement and procedure‐related costs.

The simulation analysis also illustrates how changes in the distribution of surgical approaches could influence institutional financial performance. In scenarios dominated by open or laparoscopic approaches, the annual gross profit remained relatively stable. However, increasing the proportion of robotic procedures progressively reduced the annual gross profit under the current reimbursement structure, highlighting the financial sensitivity of hospital income to the procedural mix when high‐cost technologies are introduced without proportional reimbursement adjustments.

### 3.3 Sensitivity Analysis of Revenue and Cost Parameters

Sensitivity analyses were performed to examine the robustness of gross profit estimates by varying the major revenue and cost parameters by ± 20% from the baseline values for both robotic DG and robotic TG. In both procedures, changes in surgical procedure fees and material costs produced larger variations in gross profit than changes in maintenance or labour costs (Supporting Information S1: Figure S2).

Among the evaluated parameters, changes in surgical procedure fees produced the largest variation in gross profit, followed by changes in material costs. In contrast, variations in labour or robot maintenance costs had comparatively smaller effects on overall financial outcomes within the tested range. Although robotic TG remained positive across most tested scenarios, its gross profit margin remained substantially lower than that of open TG. These findings suggest that reimbursement levels and consumable costs are the principal determinants of institutional profitability in robotic gastrectomy.

## 4 Discussion

Robotic surgery represents a major technological investment within modern surgical care. When reimbursement is only partially adjusted for new technologies, hospitals may experience financial pressure despite policy support for innovation. Understanding how reimbursement design aligns with procedure‐specific cost structures is therefore important for institutional financial management.

Although this study was conducted within the Japanese national health insurance system, the findings have broader relevance to international discussions regarding the pragmatic adoption of high‐cost surgical technologies. Publicly financed healthcare systems increasingly face the challenge of balancing rapid technological innovation with institutional financial constraints. Robotic surgery is a typical example of such innovation, in which the clinical advantages of advanced surgical platforms must be weighed against their substantial capital and operational costs. Our findings suggest that when reimbursement increments do not adequately reflect the cost structure of robotic procedures, hospitals may face financial disincentives despite the clinical adoption of the technology. This issue is not unique to Japan and has been discussed in various healthcare systems, highlighting the importance of aligning reimbursement design with real‐world cost structures to ensure the pragmatic implementation of advanced surgical technologies.

In this single‐institution analysis, robotic gastrectomy demonstrated heterogeneous financial performance across procedures. Although robotic total gastrectomy (TG) remained profitable, robotic distal gastrectomy (DG) generated negative gross profit under the current reimbursement framework. These findings illustrate that uniform reimbursement increments may not adequately account for procedure‐level variations in cost structures. Such misalignment may influence institutional adoption strategies and create unintended financial pressure within hospitals operating under fixed or tightly regulated reimbursement systems. Interpretation of the TG findings should be made cautiously because no laparoscopic TG cases were available during the study period. Therefore, the present analysis does not provide a complete comparative economic assessment across all minimally invasive approaches for TG.

One of the main factors influencing the profitability of robot‐assisted gastric cancer surgery was the imbalance between procedure‐related costs and incremental reimbursement. Although robotic gastrectomy incurs higher procedure fees than laparoscopic surgery, the increase in total reimbursement was limited relative to the additional material and maintenance costs incurred. This imbalance was particularly apparent for the DG, in which higher procedure‐related costs were not sufficiently offset by increased reimbursement. Our findings are consistent with previous hospital‐based studies conducted within Japan's public healthcare system, which demonstrated that robotic gastrectomy generally yields lower gross profit margins than laparoscopic surgery despite higher reimbursement [4]. In that study, robotic TG remained profitable, whereas robotic DG and PG showed substantially lower profitability, with PG resulting in a negative gross profit. These findings are broadly consistent with our observation that robotic TG was relatively less affected, whereas robotic DG demonstrated a negative gross profit. Minor differences in the absolute profit estimates are likely attributable to institutional variations in procurement costs, maintenance cost allocation, and consumable use, including differences in reconstruction methods for DG and PG. Similar reductions in hospital profitability associated with robotic surgery have also been reported outside Japan, including a single‐institution study comparing robotic and laparoscopic rectal resection in South Korea [20]. Overall, these findings suggest that procedure‐specific reimbursement often fails to fully offset the additional costs of robotic surgery across different healthcare settings.

Robot maintenance costs represent fixed institutional expenses that are typically allocated on a per‐case basis. In the supplementary scenario analysis, an annual volume of 400 robotic procedures was assumed, reflecting an optimistic utilization scenario for robotic systems installed at our institution. As shown in the scenario analysis, increasing the annual number of robotic procedures reduced the maintenance cost per case; however, even under an assumed higher annual case volume, the overall profitability of robot‐assisted DG did not change substantially. This finding suggests that factors other than case volume, including reimbursement structure and material costs, contribute to differences in gross profit across surgical approaches.

Sensitivity analyses demonstrated that gross profit was more sensitive to changes in surgical procedure fees and material costs than to changes in robot maintenance or labour costs. Similar trends were observed in both distal and total gastrectomy analyses. These findings suggest that variations in procedure‐related reimbursement and consumable usage may have a greater influence on financial outcomes than institutional factors, such as staffing or maintenance allocation.

This study has several limitations. First, this was a single‐institution analysis, and cost structures may differ across hospitals depending on institutional policies, contractual arrangements, and case mix differences. However, single‐institution analyses may also provide important insights into the real‐world financial implications of robotic surgery because institutional accounting data allow for a detailed assessment of procedure‐level costs that are often unavailable in large administrative databases. Second, proximal gastrectomy was excluded from the comparative profitability analyses because no open or laparoscopic cases were performed during the study period, limiting procedure‐specific comparisons. Third, cost estimates were based on institutional accounting data and representative cases, and variations related to operative complexity or reconstruction methods were not completely captured. Fourth, postoperative complication‐related costs were not incorporated into the gross profit calculation; therefore, the present findings reflect the intraoperative financial balance rather than the total episode‐of‐care cost.

In conclusion, this study provides a procedure‐specific description of the financial characteristics of robotic surgery for gastric cancer in a publicly financed healthcare system. These findings demonstrate that the procedure‐specific gross profit patterns differed substantially across surgical approaches under the current reimbursement framework. Even in scenarios of increased robotic utilization, structural imbalances between reimbursement and procedure‐related costs may persist. These results highlight the importance of aligning the reimbursement design with real‐world cost structures when introducing high‐cost surgical technologies. Such evidence may inform future discussions on reimbursement policies and the institutional adoption of surgical innovation within publicly financed healthcare systems worldwide.

## Author Contributions

**Yoshimasa Akashi:** conceptualisation, methodology, investigation, writing – original draft preparation. **Koichi Ogawa:** writing – review and editing. **Yohei Owada:** writing – review and editing. **Tsuyoshi Enomoto:** writing – review and editing. **Kinji Furuya:** writing – review and editing. **Kazuhiro Takahashi:** writing – review and editing. **Osamu Shimomura:** writing – review and editing. **Yoshihiro Miyazaki:** writing – review and editing. **Tatsuya Oda:** supervision.

## Funding

The authors have nothing to report.

## Conflicts of Interest

The authors declare no conflicts of interest.

## Supporting information


Supporting Information S1

## Data Availability

The data that support the findings of this study are available on request from the corresponding author. The data are not publicly available due to privacy or ethical restrictions.
