# Supplementary material for: Reimbursement Design and Procedure‐Level Gross Profit of Robot‐Assisted Gastrectomy in a Publicly Financed Health System: Evidence From Japan
Source: Int J Health Plann Manage. 2026 Aug 17;41(6):1270–6. doi: 10.1002/hpm.70127 (PMC13730257; doi:10.1002/hpm.70127)
Supplement: Supplementary file 1 [file HPM-41-1270-s001.docx]

**Supplementary Online material – Index**

**Supplementary Tables**

Table S1. Distribution of reconstruction methods for distal gastrectomy

Table S2. Representative procedural material-utilization patterns used for material-cost estimation

**Supplementary Figures**

Figure S1. Robot maintenance cost per procedure according to the annual robotic procedure volume

Figure S2. Sensitivity analysis of revenue and cost parameters for robotic gastrectomy

**Table S1. Distribution of reconstruction methods for distal gastrectomy**

| **Surgical Approach (n)** | **Billroth I** | **Roux-en-Y** |
| --- | --- | --- |
| Open DG (n=6) | 0 | 6 (100%) |
| Laparoscopic DG (n=12) | 3 (25%) | 9 (75%) |
| Robotic DG (n=18) | 4 (22.2%) | 14 (77.8%) |

*DG; distal gastrectomy*

*No other reconstruction methods were performed.*

**Table S2. Representative procedural material-utilization patterns used for material-cost estimation.**

Material-utilization patterns were defined by the operating surgeons based on routine clinical practice at our institution and were used for procedural cost estimation. Values indicate the number of consumable items used per representative procedure and do not represent exact case-level utilization. Institutional purchase prices were applied separately for gross-profit calculations but are not shown because procurement costs varied according to institutional purchasing contracts.

**Figure S1. Robot maintenance cost per procedure according to the annual robotic procedure volume**

The curve depicts the relationship between the annual robotic procedure volume (X-axis) and the maintenance cost per robotic procedure (Y-axis). In 2024, 346 robotic procedures were performed, resulting in a maintenance cost of approximately ¥311,000 per procedure. In a scenario analysis, assuming 400 robotic surgeries per year (200 per robot annually with two robots) while keeping the annual maintenance costs constant, the maintenance cost per procedure was approximately ¥270,000.

**Figure S2. Sensitivity analysis of revenue and cost parameters for robotic gastrectomy**

a) **Distal gastrectomy**

b) **Total gastrectomy**

Sensitivity analysis was performed by varying the major revenue and cost parameters by ±20% from the baseline values based on 2024 institutional accounting estimates. The figure illustrates the relative changes in gross profit associated with variations in each parameter. The horizontal dashed line represents the reference point of the zero gross profit. The overall interpretation remained stable across the tested parameter ranges, with surgical procedure fees and material costs exerting the greatest influence on gross profit estimates.
